# A Python package for parsing, validating, mapping and formatting sequence variants using HGVS nomenclature

**DOI:** 10.1093/bioinformatics/btu630

**Published:** 2014-09-30

**Authors:** Reece K. Hart, Rudolph Rico, Emily Hare, John Garcia, Jody Westbrook, Vincent A. Fusaro

**Affiliations:** ^1^Invitae Inc., San Francisco, CA 94107 and ^2^23andMe Inc., Mountain View, CA 94043, USA

## Abstract

**Summary:** Biological sequence variants are commonly represented in scientific literature, clinical reports and databases of variation using the mutation nomenclature guidelines endorsed by the Human Genome Variation Society (HGVS). Despite the widespread use of the standard, no freely available and comprehensive programming libraries are available. Here we report an open-source and easy-to-use Python library that facilitates the parsing, manipulation, formatting and validation of variants according to the HGVS specification. The current implementation focuses on the subset of the HGVS recommendations that precisely describe sequence-level variation relevant to the application of high-throughput sequencing to clinical diagnostics.

**Availability and implementation:** The package is released under the Apache 2.0 open-source license. Source code, documentation and issue tracking are available at http://bitbucket.org/hgvs/hgvs/. Python packages are available at PyPI (https://pypi.python.org/pypi/hgvs).

**Contact:** reecehart@gmail.com

**Supplementary information:** Supplementary data are available at *Bioinformatics* online.

## 1 INTRODUCTION

As high-throughput sequencing becomes commonplace in the investigation and diagnosis of disease, it is essential that communicating variants from sequencing projects to the scientific community and from diagnostic laboratories to health-care providers is easy and accurate. The Human Genome Variation Society (HGVS) mutation nomenclature recommendations (Taschner and den Dunnen, 2011) are widely endorsed by professional organizations, mandated by numerous journals and displayed by databases and tools. The HGVS recommendations—originally devised to standardize the representation of variants discovered before the advent of high-throughput sequencing—are now approved by the HGVS and continue to evolve under the auspices of the Human Variome Project. The continual evolution of HGVS guidelines makes the nomenclature difficult to understand and to use for experts and non-experts alike, often resulting in incorrect usage and potential clinical interpretation errors.

We sought a software library for manipulating HGVS variants that was appropriate for clinical diagnostics. Specifically, we required the following functionality: (i) We must be able to process patient variants locally (i.e. not sent to a remote site); (ii) we must be able to audit, extend and control updates of the source code and data; (iii) we must be able to map variants in regions of genome-transcript discrepancies, particularly indels. Mutalyzer (Wildeman *et al.*, 2008) provides a Web interface and Web services for constructing, validating and transforming sequence variants, but is not available for local installation. Another Python library (https://github.com/counsyl/hgvs), similar in spirit to the one we present here, uses a regular expression-based parser and relies on exon structures derived from BLAT alignments (Kent, 2002). Although other packages accept or generate HGVS-formatted variants, such as snpEff (Cingolani *et al.*, 2012) and VEP (Mclaren *et al.*, 2010), they are not intended for use as a software library.

Here we present an open-source Python library for parsing, mapping, validating and formatting sequence variation according to the HGVS guidelines. The library features a parser based on a Parsing Expression Grammar and a variant mapper that accommodates insertion/deletion discrepancies between reference genomic sequences and transcripts that confound most existing tools.

## 2 PACKAGE OVERVIEW

The *hgvs* Python package comprises six key components: (i) object models for representing components of HGVS-formatted variants; (ii) a parser that generates an object representation from an HGVS-formatted string; (iii) formatting tools that generate an HGVS-formatted string from an object representation; (iv) mapping classes that transform variants between genomic, CDS and protein representations; (v) validation tools that ensure conformance to HGVS guidelines; (vi) an interface for defining external data sources required for validation and mapping.

**Fig. 1.**
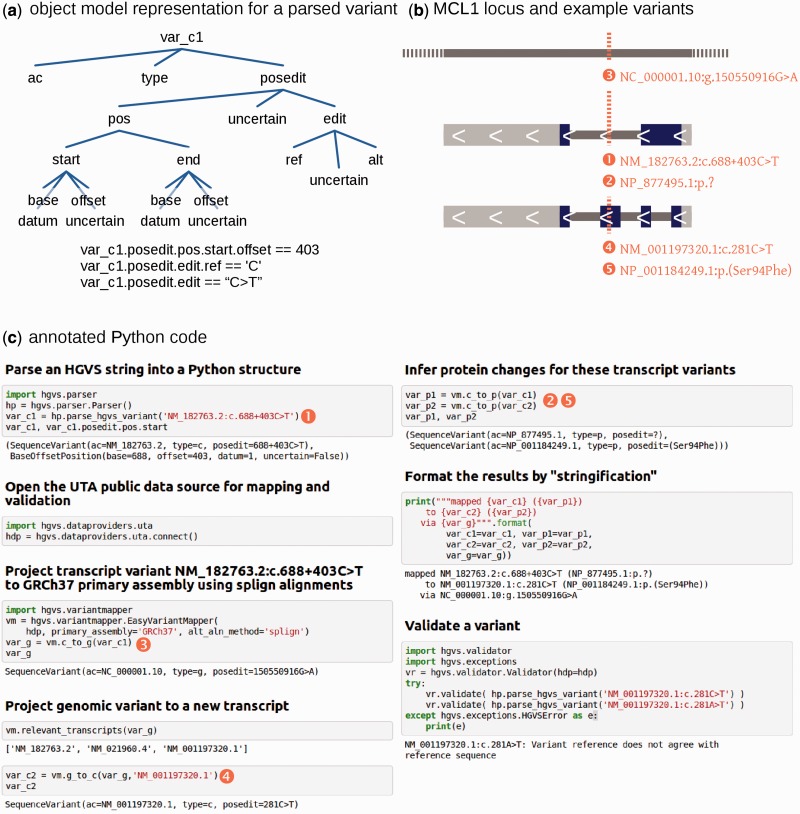
Using the *hgvs* package to project a variant in MCL1 from one transcript to another via GRCh37 chromosome 1. (**a**) An object representation of the result of parsing ‘NM_182763.2:c.688+403C>T’. Selected attributes are shown beneath. (**b**) A diagram of the MCL1 locus with five representations of a single variant. (**c**) Python code that demonstrates parsing, mapping between sequences, formatting and validating. Gray outline boxes enclose input, and the results appear immediately beneath. Circled numbers indicate a correspondence between the variants in (**a**) and code in (**c**). An SNV❶, originally reported in literature as NM_182763.2:c.688+403C>T (rs201430561), is projected onto chromosome 1 as variant ❸, and then projected to an alternative transcript as variant. The inferred protein❹ changes of variants ❶ and ❹ are shown as protein variants❷ and ❺. The results are formatted by ‘stringifying’ them using standard Python printing commands. Validation for a valid variant (281C>T; no error generated) and an error for an invalid variant (281A>T) are shown

The core of the *hgvs* package is a set of object models that provide a foundation for developers to reference any component or property of the HGVS syntax. For example, a SequenceVariant consists of a sequence accession, a sequence type and PosEdits, which represent a set of individual changes to a sequence. A PosEdit is composed of positions, such as a BaseOffsetPosition for a CDS (c.) SequenceVariant with intronic variants, and an Edit for Single Nucleotide Variant (SNV), del, ins, delins, duplications and repeats. Figure 1a shows an example object representation. For a full list of object classes, readers should consult the source code.

The HGVS parser presented here is based on a parsing expression grammar and was inspired by previous work (Laros *et al.*, 2011). There are 127 parsing rules to cover DNA, RNA, CDS and protein parsing. Any rule may be invoked to parse components of valid HGVS strings into an appropriate type. For example, the c_edit rule may be used to parse an HGVS substring such as ‘c.688+403C>T’. Following Python conventions, formatting is implemented using the str() method of each class.

The *hgvs* package requires sequence data and exon structures to map variants between the genome and transcript coordinates, to infer protein sequence changes from transcripts and to validate variants. The data provider interface declares seven methods required to support *hgvs* functionality; developers may implement a subclass of the abstract interface to use data from other sources. The *hgvs* package includes a concrete implementation based on the publicly available Universal Transcript Archive (information about UTA is available with package documentation).

In addition to the syntactic validation provided during parsing, the *hgvs* package includes validation tools to ensure that a SequenceVariant object conforms to the HGVS guidelines and to catch common errors. For performance reasons, the Validator distinguishes *intrinsic* and *extrinsic* validation: intrinsic validation asserts internal correctness of the object, such as requiring that the start position is less than or equal to the end position or that the length of the location range specified for an insertion is one, and extrinsic validation invokes external data for validation, such as verifying that the reference sequence specified in the variant matches that from a source database.

The *hgvs* package provides tools to transform (‘map’) variants between genomic (g.), mRNA (r.), CDS (c.) and protein (p.) variants. Of particular note is the implementation of an indel aware mapper that correctly accounts for insertions and deletions in transcripts with respect to a genomic reference. These discrepancies occur owing to natural polymorphisms and sequencing errors, and occur in ∼1.6% of current RefSeq transcripts (Garla *et al.*, 2011). The package also includes a transcript liftover tool to migrate variants between different transcripts. When used in conjunction with UTA, liftover may also be performed between the same RefSeq transcript aligned to a genomic reference by Splign (Kapustin *et al.*, 2008) and BLAT (Kent, 2002). Splign and BLAT provide substantially different exon structures for ∼2.7% of RefSeq transcripts.

## 3 TESTING, VALIDATION AND LIMITATIONS

Reliable and robust variant manipulation is an essential goal of this work. The *hgvs* package implements extensive automated tests that are run on every commit made to the *hgvs* code, and the test results are publicly accessible. Unit tests, which validate low-level functionality, are available for nearly all code in the package. Functional tests, which verify parsing, formatting, transformation and validation, are extensive. In particular, 163 manually curated mappings between g., c. and p. representations in problematic genes were developed by geneticists and curators. Additional functional tests include >20 000 intronic and exonic single nucleotide variants, >2000 deletions, insertions and deletion-insertions variants and 11 duplications in 54 genes that exercise a variety of transcript features, such as strand, CDS start not in exon 1 and genome-transcript indels.

In addition to the above automated tests, a comparison of genome-to-transcript transformations generated by the *hgvs* package and Mutalyzer’s batch positionConverter tool using 110 125 genomic variants in 57 ACMG ‘Must Report’ genes (Green *et al.*, 2013) showed >99.9% concordance (Supplementary data). Mismatches occurred only in transcripts that have indels in the genome-transcript alignment. Because *hgvs* uses an indel aware mapper and Mutalyzer does not (Peter Taschner, personal communication), these differences are expected and highlight an advantage of the library presented here.

Like the HGVS recommendations, the *hgvs* package presented here is a work in progress. The package does not yet implement the full recommendations: important limitations, which are detailed in the issue tracker, include lack of variant canonicalization, the inability to represent compound, mosaic and chimeric variants and lack of support for inversions. Patches and pull requests are welcome.

## 4 AVAILABILITY, INSTALLATION AND USE

The *hgvs* source code, comprehensive documentation, examples, installation instructions, issue tracking, test results and mailing list are available via the BitBucket repository. In addition, the package is also available at PyPI; with modern versions of Python, installation involves simply typing ‘pip install hgvs’.

Figure 1 illustrates the four primary functions provided by the *hgvs* package—parsing, formatting, mapping and validating. A variant in MCL1, NM_182763.2:c.688+403C>T (rs201430561), is parsed into a variant object that exposes variant components as Python properties. The variant is then projected onto NM_001197320.1 via GRCh37 and a splign-based exon alignment. A validation error is demonstrated with NM_001197320.1:c.281A>T, which specifies an incorrect reference nucleotide.

*Conflict of interest:* All authors are employed by and have equity in the company that sponsored this work.

## Supplementary Material

Supplementary Data
