# Supplementary material for: A Python package for parsing, validating, mapping and formatting sequence variants using HGVS nomenclature
Source: Bioinformatics. 2014 Sep 30;31(2):268–70. doi: 10.1093/bioinformatics/btu630 (PMC4287946; doi:10.1093/bioinformatics/btu630)
Supplement: Supplementary Data [file supp_31_2_268__index.html]

A Python package for parsing, validating, mapping and formatting sequence variants using HGVS nomenclature — A Python package for parsing, validating, mapping and formatting sequence variants using HGVS nomenclature — A Python package for parsing, validating, mapping and formatting sequence variants using HGVS nomenclature — Supplementary Data 

# A Python package for parsing, validating, mapping and formatting sequence variants using HGVS nomenclature

## Supplementary Data

files

**Files in this Data Supplement:**

- Supplementary Data - zip file
