# Supplementary material for: A Python package for parsing, validating, mapping and formatting sequence variants using HGVS nomenclature
Source: Bioinformatics. 2014 Sep 30;31(2):268–70. doi: 10.1093/bioinformatics/btu630 (PMC4287946; doi:10.1093/bioinformatics/btu630)
Supplement: Supplementary file 2 [file supp_btu630_Supplemental_Data.zip › Supplemental_Data_1.odt]

A Python Package for Parsing, Validating, Mapping, and Formatting Sequence Variants Using HGVS Nomenclature

Hart, RK, et al.

Supplemental Data 1: Comparison of genome-to-transcript mapping by Mutalyzer and hgvs

Code and documentation regarding this comparison are available in the hgvs source package at 

[https://bitbucket.org/hgvs/hgvs/src/cdd77cc0c47ffdcdd7a1f4a4ed22c5f0905c86e1/misc/mutalyzer-comparison/?at=default](https://bitbucket.org/hgvs/hgvs/src/cdd77cc0c47ffdcdd7a1f4a4ed22c5f0905c86e1/misc/mutalyzer-comparison/?at=default). Source data and results are available at [https://bitbucket.org/hgvs/hgvs/downloads](https://bitbucket.org/hgvs/hgvs/downloads).

Objective

Compare the transformation of genomic variants in 57 ACMG “Must Report” genes to transcript variants using Mutalyzer's batch Position Converter and the hgvs package.

Method

dbSNP 137 variants were collected from the SNP_HGVS table in BioQ ([https://bioq.saclab.net/query/downloads.php?db=bioq_dbsnp_human_137](https://bioq.saclab.net/query/downloads.php?db=bioq_dbsnp_human_137)). SNP records in 57 ACMG genes (Green et al.)⁠ were selected from syntactically valid SNP records. Some records contained invalid HGVS strings, such as missing reference alleles and multinucleotide alleles used with '>', and were discarded. The ACMG must report genomic variants were converted to transcript variants using Mutalyzer's Position Converter batch interface ([https://mutalyzer.nl/batchPositionConverter](https://mutalyzer.nl/batchPositionConverter)) on Aug 18 and with the hgvs package (version 0.3.3). For a given genomic variant, each tool returned zero or more transcript (c.) variants depending on the number of transcripts that overlap the genomic variant. Transcript variants returned by the two tools were compared when the transcript accessions (including version) matched. A transcript variant pair were deemed to match when the strings were identical or substantively equivalent, such as NM_001258274.1:c.1161_1174-3del844insGGAAA (returned by hgvs) and NM_001258274.1:c.1161_1174-3delinsGGAAA (returned by Mutalyzer) which differ by the presence of the deletion length. Mismatches were classified based on whether the specified transcript contains an indel with respect to the GRCh37 primary assembly.

Results 

129,556 distinct genomic variants were selected from BioQ, processed separately with Mutalyzer's Position Converter batch interface and hgvs, and compared. Of these, 18,460 had empty results by both tools. An additional 971 genomic variants had results from Mutalyzer but not from hgvs. Based on a sampling of 556 transcripts used by Mutalyzer, the differences between the tools occur for a variety of reasons including transcript obsolescence, alignment differences, and non-existence in UTA, which does not have XM transcripts. The remaining 110,125 genomic variants generated 263,351 transcript variants pairs, of which 263,170 (>99.9%) matched. There were 181 transcript variant pair mismatches in 16 transcripts, all of which contain an indel in the genome-transcript alignment.

| number of genomic variants | description |
| --- | --- |
| 129,556 | dbSNP 137 variants on the GRCh37 primary assembly submitted to both tools |
| 18,460 | variants with empty results by both tools |
| 971 | variants with results from Mutalyzer but not from hgvs |
| 110,125 | 263,351 transcript variant pairs |
| 110,067 | 263,170 matching transcript variant pairs |
| 0 | 0 c. variant mismatches in transcripts without genome-transcript alignment indels |
| 58 | 181 c. variant mismatches in transcripts with genome-transcript alignment indels |

Conclusion

Mutalyzer and hgvs return nearly identical results – >99.9% concordance for all transcript variant pairs compared – for the transformation of genomic variants to transcript variants in 57 ACMG “must report” genes. All 181 mismatches occur in 16 transcripts that have indels in the genome-transcript alignment. Because hgvs uses an indel-aware mapper and Mutalyzer does not (Peter Taschner, personal communication, May 2014), these differences are expected.
